# First hydrogen-bonded adduct of sterically hindered 2-*tert*-butyl-4-methyl­phenol (TBMP) with 1,3,6,8-tetra­aza­tri­cyclo­[4.4.1.1^3,8^]dodecane (TATD) *via* coupling of classical hydrogen bonds and C—H⋯π non-covalent inter­actions

**DOI:** 10.1107/S2056989022004972

**Published:** 2022-05-17

**Authors:** Augusto Rivera, Jaime Ríos-Motta, Michael Bolte

**Affiliations:** a Universidad Nacional de Colombia, Sede Bogotá, Facultad de Ciencias, Departamento de Química, Cra 30 No. 45-03, Bogotá, Código Postal 111321, Colombia; b Institut für Anorganische Chemie, J. W. Goethe-Universität Frankfurt, Max-von Laue-Str. 7, 60438 Frankfurt/Main, Germany; Universität Greifswald, Germany

**Keywords:** crystal structure, co-crystalline adduct, hydrogen bonding, C—H⋯π inter­actions, TBMP, TATD

## Abstract

The title compound, C_8_H_16_N_4_·2C_11_H_16_O, was synthesized from the corres­ponding sterically crowded phenol by treatment with the aminal cage polyamine. Single-crystal X-ray diffraction structural analysis revealed the three-mol­ecule aggregate to crystallize in the monoclinic space group *P*2/*c* with one half of a 1,3,6,8-tetra­aztri­cyclo­[4.4.1.1^3,8^]dodecane (TATD) mol­ecule and one 2-*tert*-butyl-4-methyl­phenol mol­ecule per asymmetric unit. The crystal structure features inter­molecular O—H⋯N and C—H⋯O hydrogen bonds, as well as inter­molecular C—H⋯π inter­actions.

## Abstract 2

Hydrogen bonding links the donor alcohol functional groups of two 2-*tert*-butyl-4-methyl­phenol mol­ecules to the central acceptor polyamine aminal cage TATD yielding the three mol­ecule adduct, half of which comprises the asymmetric unit in this crystal structure.

## 1. Chemical context

Co-crystals of phenols with various nitro­gen bases are model systems often used for studying the nature of the hydrogen bond (Majerz *et al.*, 2007 ▸). In this context, not only the initial formation of a hydrogen-bonded adduct was investigated between a Mannich preformed reagent and the phenolic substrate (Burckhalter & Leib, 1961 ▸), but also the great inter­est in and chemical importance of the amino­alkyl­ation of aromatic substrates *via* the Mannich reaction was addressed (Tramontini *et al.*, 1988 ▸). For a long time we have directed continuing efforts to the systematic study of hydrogen bonding and other non-covalent inter­actions of phenols with aminal cages (preformed Mannich bases) (Rivera *et al.*, 2007 ▸, 2015*a* ▸,*b* ▸, 2017*a* ▸,*b* ▸, 2019 ▸). Herein we report the mechanochemical preparation and crystal structure of the title adduct prepared by mixing in an agate mortar the sterically hindered 2-*tert*-butyl-4-methyl­phenol (TBMP) with 1,3,6,8-tetra­azatri­cyclo­[4.4.1.1^3,8^]dodecane (TATD) in a 2:1 ratio. The crystallographic information available for pure 2-*tert*-butyl-4-methyl­phenol (Beckmann *et al.*, 2004 ▸) does not report O—H⋯O hydrogen bonds, which are commonly found in the crystal structures of alcohols, suggesting that the alcohol is sterically protected. The reaction of TBMP with TATD, in notable contrast to this, proceeds cleanly to give the title O—H⋯N hydrogen-bonded adduct exclusively. A search of the Cambridge Structural Database (version 5.42; Groom *et al.*, 2016 ▸) for crystal structures containing hydrogen-bonded TBMP co-crystals with a hydrogen-bond acceptor resulted in zero hits, emphasizing the general rarity of this observation. The resultant crystal structure reported here also exhibits C—H⋯O hydrogen-bonding inter­actions, which constitute a fundamental force in maintaining crystal and three-dimensional chemical structures in chemistry and biology (Wang *et al.*, 2019 ▸). 
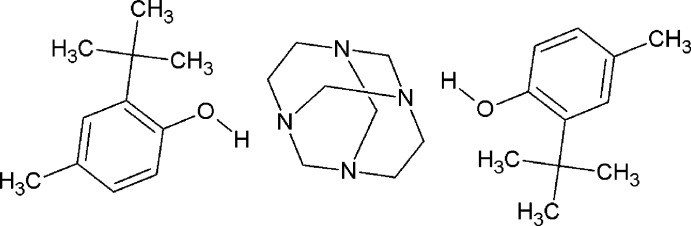


## 2. Structural commentary

**Figure 1:**
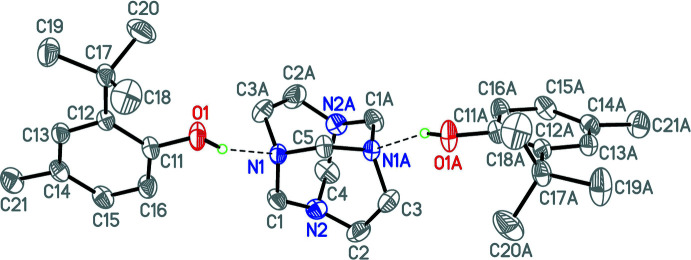
A view of the mol­ecular structure of the title compound, showing the atom-labelling scheme, with displacement ellipsoids drawn at the 50% probability. H atoms bonded to C atoms are omitted for clarity. Hydrogen bonds are drawn as dashed lines. Atoms labelled with the suffix A are generated using the symmetry operator (−*x*, *y*, −*z* + 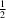 ).

The title compound crystallizes in the monoclinic space group *P*2*/c*. The asymmetric unit comprises one half of a 1,3,6,8-tetra­azatri­cyclo­[4.4.1.1^3,8^]dodecane (TATD) mol­ecule and one 2-*tert*-butyl-4-methyl­phenol (TBMP) mol­ecule held together by one inter­molecular O—H⋯N hydrogen bond [O⋯N = 2.8534 (15) Å; O—H⋯N = 161.6 (17)°; Table 1 ▸]. The complete adduct is generated by symmetry by a crystallographic twofold rotation axis, resulting in *C*2 symmetry for the three-mol­ecule aggregate (Fig. 1 ▸). Apart from the two neutral inter­molecular O—H⋯N bonds in the three-mol­ecule arrangement, as indicated by a *PLATON* analysis (Spek, 2020 ▸), there are four non-classical intra­molecular C—H⋯O hydrogen bonds between the TBMP phenol oxygen atoms and the *ortho tert*–butyl C—H bonds (two for each phenol oxygen atom O1; methyl group atoms C18—H18*B* and C20—H20*A*; geometric details are given in Table 1 ▸).

The –OH group is not perfectly co-planar with the benzene ring with a C16—C11—O1—H1 torsion angle of 18.0°. This angle differs from the corresponding more acute torsion angles in free 2-*tert*-butyl-4-methyl­phenol (0.73 and −0.36°; Beckmann *et al.*, 2004 ▸) and other related sterically very congested phenols (Lutz & Spek, 2005 ▸). The observed C11—O1 bond length [1.376 (2) Å] is in a good agreement with the mean value of 1.377 Å reported for 2-*tert*-butyl-4-methyl­phenol (Beckmann *et al.*, 2004 ▸).

The C—N1 bond lengths of the nitro­gen atom, which is engaged in the inter­molecular hydrogen bond to TBMP, are slightly elongated at 1.476 (2) Å (N1—C1), 1.469 (2) Å (N1—C3) and 1.468 (2) Å (N1—C5) compared to the mean value of 1.458 Å reported for the free aminal cage structure (Rivera *et al.*, 2014 ▸) and compared to the C—N2 bond lengths here [1.452 (2) Å (N2—C1), 1.456 (2) Å (N2—C2), and 1.462 (2) Å (N2—C4)]. This indicates that the formation of the inter­molecular hydrogen bonds in the title compound affects the distribution of electron density around this hydrogen-bonded nitro­gen centre, resulting in an impact on the respective CH_2_—N single bonds in the heterocyclic cage system.

## 3. Supra­molecular features

**Figure 2:**
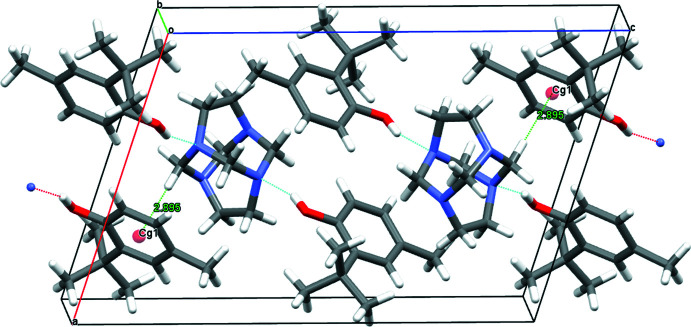
The crystal packing of the title compound viewed roughly along the *b*-axis direction, showing the inter­molecular O—H⋯N hydrogen bonds and selected C—H⋯π inter­actions.

**Figure 3:**
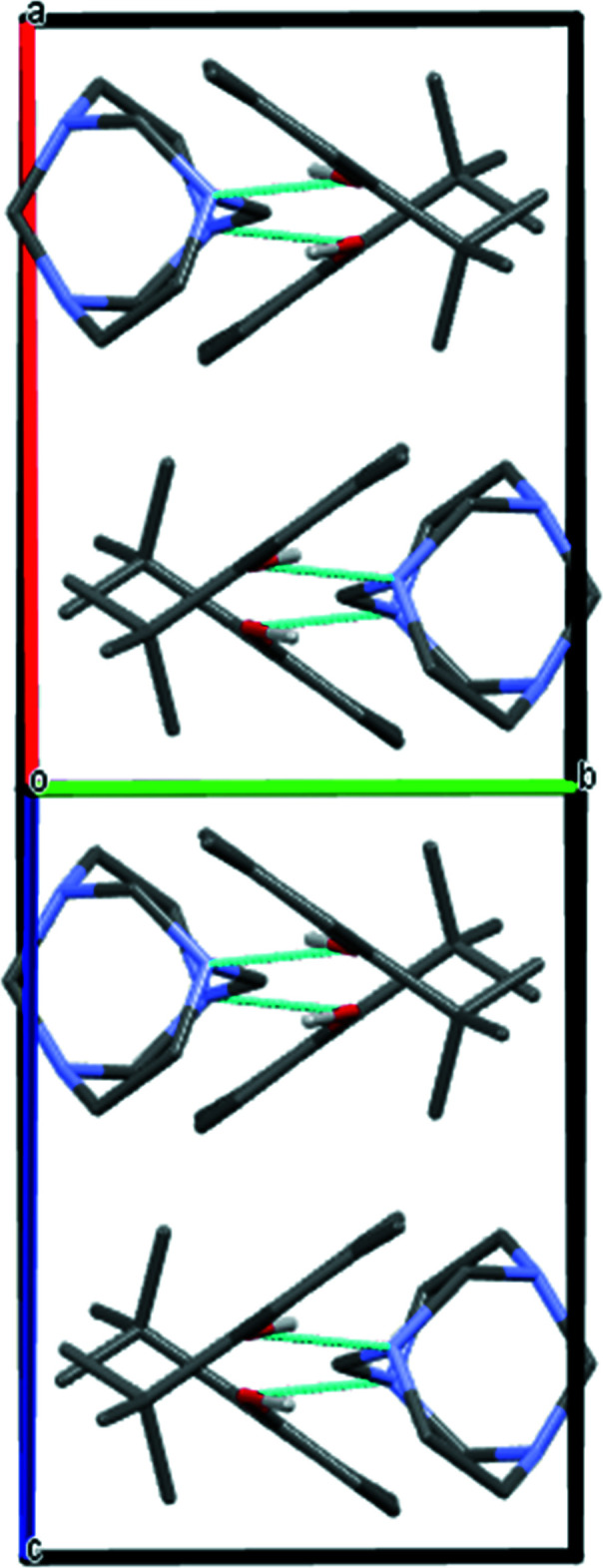
A partial packing diagram viewed along [101] direction. Dashed lines indicate the inter­molecular O—H⋯N hydrogen bonds. Only H atoms involved in the hydrogen bonds are shown for clarity.

The most prominent supra­molecular feature in this crystal structure is the formation of the expected three-mol­ecule aggregate sustained by two hy­droxy-O—H⋯N hydrogen bonds (Fig. 2 ▸). In the crystal packing, roughly in the *a*-axis direction, adjacent aggregates are linked by C—H⋯π inter­actions with a C—H⋯*Cg* distance of 3.851 (2) Å and a C—H⋯*Cg* angle of 163°, (Table 1 ▸). The C—H⋯π inter­action is facilitated between one methyl­ene group (C1—H1*A*) and a symmetry-derived ring (C11–C16; symmetry code: −*x* + 1, −*y* + 1, −*z* + 1). These non-covalent inter­actions lead to the formation of a crystal packing pattern in which the phenol mol­ecules are arranged in an alternating fashion, as is evident when viewed along the [101] direction (Fig. 3 ▸).

## 4. Database survey

Using the Cambridge Structural Database (CSD, Version 5.42, September 2021 update; Groom *et al.*, 2016 ▸), a search for the title compound structure and names used in this article was conducted with *CONQUEST* (version 2021.2.0; Bruno *et al.*, 2002 ▸). The crystal structures of both 2-*tert*-butyl-4-methyl­phenol (TBMP; Beckmann *et al.*, 2004 ▸) and 1,3,6,8-tetra­aza­tri­cyclo­[4.4.1.1^3,8^]dodecane (TATD; Rivera *et al.*, 2014 ▸) are already known (refcodes: PAGMEQ and TAZTCD). 2-*tert*-Butyl-4-methyl­phenol crystallizes with two mol­ecules in the asymmetric unit, which exhibit non-classical intra­molecular C—H⋯O hydrogen bonds similar to what is found in the adduct structure reported here, plus weak inter­molecular O—H⋯π inter­actions. Tetra­aza­tri­cyclo­[4.4.1.1^3,8^]dodecane crystallizes with one quarter of a mol­ecule in the asymmetric unit. There are no significant differences in the metrical parameters between the structure of the title co-crystal and the singly crystallized entities except for the C—N distances discussed above (section 2).

Co-crystals of tetra­aza­tri­cyclo­[4.4.1.1^3,8^]dodecane have already been reported, *i.e.* with 3-nitro­phenol (Rivera *et al.*, 2019 ▸), 4-iodo­phenol (Rivera *et al.*, 2017*a* ▸), 4-chloro-3,5-di­methyl­phenol (Rivera *et al.*, 2015*a* ▸), hydro­quinone (Rivera *et al.*, 2007 ▸), and 4-bromo­phenol (Rivera *et al.*, 2015*b* ▸) (refcodes: HOXGUZ, JELVII, QUFROA, WEXQIA, XULKOG).

In addition, one crystal structure with a singly protonated tetra­aza­tri­cyclo­[4.4.1.1^3,8^]dodecane was determined prev­iously, namely 3,6,8-tri­aza-1-azoniatri­cyclo­[4.4.1.1^3,8^]dodecane 4-nitro­phenolate 4-nitro­phenol (Rivera *et al.*, 2017*b* ▸; refcode: REYKAK).

In another closely related adduct structure, a slightly less sterically crowded alcohol was used bearing an *iso*-propyl instead of the *tert*-butyl substituent on the aromatic ring: tris-[5-methyl-2-(propan-2-yl)phenol]1,3,5,7-tetra­aza­tri­cyclo­[3.3.1.1^3,7^]decane (Mazzeo *et al.*, 2019 ▸; refcode: WUTDUN).

## 5. Synthesis and crystallization

A mixture of 1,3,6,8-tetra­aza­tri­cyclo­[4.4.1.1^3,8^]dodecane (TATD) (1 mmol) and 2-*tert*-butyl-4-methyl­phenol (TBMP) (2 mmol) was ground using a mortar and pestle at room temperature for 15 min. Completion of the reaction was monitored by TLC. The mixture was recrystallized from *n*-hexa­ne:chloro­form (8:2) solution to obtain colourless crystals suitable for X-ray analysis, m.p. = 374–375 K. (yield: 85%).

## 6. Refinement

The structure of the title compound had been previously deposited by us and was thereby reported as a Private Communication (Bolte *et al.*, 2021 ▸, refcode EWICAR). Crystal data, data collection and structure refinement details are summarized in Table 2 ▸. The oxygen-bound hydrogen atom was found and refined isotropically without restraints or constraints. Other hydrogen atoms were generated geometrically, and refined with a riding model with C—H = 0.98 Å, *U*_iso_(H) = 1.5*U*_eq_(C) for methyl, C—H = 0.99 Å, *U*_iso_(H) = 1.2*U*_eq_(C) for methyl­ene, and C—H = 0.95 Å, *U*_iso_(H) = 1.2*U*_eq_(C) for aromatic hydrogen atoms.

## Supplementary Material

Crystal structure: contains datablock(s) I. DOI: 10.1107/S2056989022004972/yz2019sup1.cif

Structure factors: contains datablock(s) I. DOI: 10.1107/S2056989022004972/yz2019Isup2.hkl

Supporting information file. DOI: 10.1107/S2056989022004972/yz2019Isup3.cml

CCDC reference: 2092229

Additional supporting information:  crystallographic information; 3D view; checkCIF report

## Figures and Tables

**Table 1:** Hydrogen-bond geometry (Å, °) *Cg*1 is the centroid of the C11–C16 ring.

*D*—H⋯*A*	*D*—H	H⋯*A*	*D*⋯*A*	*D*—H⋯*A*
O1—H1⋯N1	0.88 (2)	2.01 (2)	2.8534 (15)	161.6 (17)
C18—H18*B*⋯O1	0.98	2.30	2.966 (2)	124
C20—H20*A*⋯O1	0.98	2.41	3.058 (3)	124
C1—H1*A*⋯*Cg*1^i^	0.98	2.90	3.851 (2)	163

Symmetry code: (i) 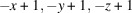 .

**Table 2:** Experimental details

Crystal data	
Chemical formula	C_8_H_16_N_4_·2C_11_H_16_O
*M*_r_	496.72
Crystal system, space group	Monoclinic, *P*2/*c*
Temperature (K)	173
*a*, *b*, *c* (Å)	11.4741 (10), 7.6770 (5), 17.2226 (14)
β (°)	108.166 (6)
*V* (Å^3^)	1441.5 (2)
*Z*	2
Radiation type	Mo *K*α
μ (mm^−1^)	0.07
Crystal size (mm)	0.28 × 0.27 × 0.11

Data collection	
Diffractometer	Stoe IPDS II two-circle
Absorption correction	Multi-scan (*X-AREA*; Stoe & Cie, 2001 ▸)
*T*_min_, *T*_max_	0.554, 1.000
No. of measured, independent and observed [*I* > 2σ(*I*)] reflections	17127, 3307, 2862
*R*_int_	0.029
(sin θ/λ)_max_ (Å^−1^)	0.653

Refinement	
*R*[*F* ^2^ > 2σ(*F* ^2^)], *wR*(*F* ^2^), *S*	0.050, 0.132, 1.05
No. of reflections	3307
No. of parameters	170
H-atom treatment	H atoms treated by a mixture of independent and constrained refinement
Δρ_max_, Δρ_min_ (e Å^−3^)	0.26, −0.19

Computer programs: *X-AREA* (Stoe & Cie, 2001 ▸), *SHELXS* (Sheldrick, 2008 ▸), *SHELXL* (Sheldrick, 2015 ▸) and *XP* in *SHELXTL-Plus* (Sheldrick, 2008 ▸).

## supplementary crystallographic information

### Crystal data

**Table:**

C_8_H_16_N_4_·2C_11_H_16_O	*F*(000) = 544
*M**_r_* = 496.72	*D*_x_ = 1.144 Mg m^−^^3^
Monoclinic, *P*2/*c*	Mo *K*α radiation, λ = 0.71073 Å
*a* = 11.4741 (10) Å	Cell parameters from 17127 reflections
*b* = 7.6770 (5) Å	θ = 3.6–27.8°
*c* = 17.2226 (14) Å	µ = 0.07 mm^−^^1^
β = 108.166 (6)°	*T* = 173 K
*V* = 1441.5 (2) Å^3^	Plate, colourless
*Z* = 2	0.28 × 0.27 × 0.11 mm

### Data collection

**Table:**

STOE IPDS II two-circle-diffractometer	2862 reflections with *I* > 2σ(*I*)
Radiation source: Genix 3D IµS microfocus X-ray source	*R*_int_ = 0.029
ω scans	θ_max_ = 27.6°, θ_min_ = 3.6°
Absorption correction: multi-scan (X-Area; Stoe & Cie, 2001)	*h* = −14→14
*T*_min_ = 0.554, *T*_max_ = 1.000	*k* = −9→9
17127 measured reflections	*l* = −22→22
3307 independent reflections	

### Refinement

**Table:**

Refinement on *F*^2^	Hydrogen site location: mixed
Least-squares matrix: full	H atoms treated by a mixture of independent and constrained refinement
*R*[*F*^2^ > 2σ(*F*^2^)] = 0.050	*w* = 1/[σ^2^(*F*_o_^2^) + (0.0663*P*)^2^ + 0.4499*P*] where *P* = (*F*_o_^2^ + 2*F*_c_^2^)/3
*wR*(*F*^2^) = 0.132	(Δ/σ)_max_ < 0.001
*S* = 1.05	Δρ_max_ = 0.26 e Å^−^^3^
3307 reflections	Δρ_min_ = −0.19 e Å^−^^3^
170 parameters	Extinction correction: SHELXL-2016/6 (Sheldrick 2015), Fc^*^=kFc[1+0.001xFc^2^λ^3^/sin(2θ)]^-1/4^
0 restraints	Extinction coefficient: 0.021 (5)
Primary atom site location: structure-invariant direct methods	

### Special details

**Table:**

Geometry. All esds (except the esd in the dihedral angle between two l.s. planes) are estimated using the full covariance matrix. The cell esds are taken into account individually in the estimation of esds in distances, angles and torsion angles; correlations between esds in cell parameters are only used when they are defined by crystal symmetry. An approximate (isotropic) treatment of cell esds is used for estimating esds involving l.s. planes.

### Fractional atomic coordinates and isotropic or equivalent isotropic displacement parameters (Å^2^)

**Table:**

	*x*	*y*	*z*	*U*_iso_*/*U*_eq_	Occ. (<1)
N1	0.55515 (9)	0.67062 (14)	0.32614 (6)	0.0269 (2)	
N2	0.41669 (11)	0.93222 (15)	0.28288 (7)	0.0334 (3)	
C1	0.47468 (13)	0.80357 (18)	0.34461 (8)	0.0332 (3)	
H1A	0.408781	0.741591	0.359229	0.040*	
H1B	0.523629	0.867230	0.394010	0.040*	
C2	0.30542 (14)	0.8720 (2)	0.22156 (10)	0.0469 (4)	
H2A	0.265533	0.973760	0.188590	0.056*	
H2B	0.248788	0.828122	0.250249	0.056*	
C3	0.31959 (13)	0.7318 (2)	0.16366 (9)	0.0397 (3)	
H3A	0.269762	0.630216	0.169446	0.048*	
H3B	0.284425	0.775888	0.107144	0.048*	
C4	0.500000	1.0300 (3)	0.250000	0.0418 (5)	
H4A	0.449637	1.106627	0.206038	0.050*	0.5
H4B	0.550362	1.106630	0.293961	0.050*	0.5
C5	0.500000	0.5738 (2)	0.250000	0.0282 (4)	
H5A	0.435761	0.497108	0.258605	0.034*	0.5
H5B	0.564238	0.497107	0.241395	0.034*	0.5
O1	0.65236 (10)	0.40303 (14)	0.44346 (6)	0.0399 (3)	
H1	0.6079 (18)	0.482 (3)	0.4109 (12)	0.053 (5)*	
C11	0.69530 (12)	0.46490 (16)	0.52226 (7)	0.0284 (3)	
C12	0.79366 (11)	0.37879 (15)	0.57928 (7)	0.0247 (3)	
C13	0.83508 (11)	0.45122 (17)	0.65783 (7)	0.0280 (3)	
H13	0.901687	0.396088	0.697378	0.034*	
C14	0.78431 (12)	0.59956 (17)	0.68142 (8)	0.0303 (3)	
C15	0.68468 (14)	0.67652 (18)	0.62418 (8)	0.0343 (3)	
H15	0.646455	0.775572	0.638772	0.041*	
C16	0.64080 (14)	0.60914 (18)	0.54571 (8)	0.0350 (3)	
H16	0.572159	0.662490	0.507184	0.042*	
C17	0.85309 (13)	0.21469 (17)	0.55726 (7)	0.0313 (3)	
C18	0.75571 (18)	0.0728 (2)	0.52590 (12)	0.0545 (5)	
H18A	0.717684	0.045775	0.568135	0.082*	
H18B	0.692811	0.114293	0.476635	0.082*	
H18C	0.794385	−0.032304	0.512895	0.082*	
C19	0.95174 (18)	0.1387 (3)	0.63136 (9)	0.0546 (5)	
H19A	0.915053	0.109330	0.674008	0.082*	
H19B	0.986240	0.033393	0.614917	0.082*	
H19C	1.017007	0.224808	0.652530	0.082*	
C20	0.91373 (19)	0.2589 (3)	0.49204 (11)	0.0565 (5)	
H20A	0.852293	0.307854	0.443928	0.085*	
H20B	0.979226	0.344302	0.514043	0.085*	
H20C	0.948459	0.152887	0.476430	0.085*	
C21	0.83767 (16)	0.6747 (2)	0.76656 (9)	0.0441 (4)	
H21A	0.895241	0.591026	0.801301	0.066*	
H21B	0.771345	0.698204	0.789736	0.066*	
H21C	0.880912	0.783389	0.763689	0.066*	

### Atomic displacement parameters (Å^2^)

**Table:**

	*U*^11^	*U*^22^	*U*^33^	*U*^12^	*U*^13^	*U*^23^
N1	0.0282 (5)	0.0284 (5)	0.0214 (5)	0.0016 (4)	0.0039 (4)	−0.0010 (4)
N2	0.0362 (6)	0.0291 (6)	0.0360 (6)	0.0066 (5)	0.0126 (5)	0.0002 (5)
C1	0.0423 (7)	0.0327 (7)	0.0270 (6)	0.0051 (6)	0.0141 (5)	−0.0016 (5)
C2	0.0303 (7)	0.0566 (10)	0.0495 (9)	0.0131 (7)	0.0064 (6)	−0.0033 (7)
C3	0.0268 (6)	0.0447 (8)	0.0407 (8)	0.0011 (6)	0.0006 (5)	−0.0006 (6)
C4	0.0581 (13)	0.0249 (9)	0.0478 (12)	0.000	0.0242 (10)	0.000
C5	0.0356 (9)	0.0236 (8)	0.0228 (8)	0.000	0.0055 (7)	0.000
O1	0.0527 (6)	0.0388 (6)	0.0200 (4)	0.0145 (5)	−0.0005 (4)	−0.0011 (4)
C11	0.0373 (7)	0.0269 (6)	0.0199 (5)	0.0007 (5)	0.0072 (5)	0.0004 (4)
C12	0.0299 (6)	0.0228 (6)	0.0215 (5)	−0.0008 (5)	0.0082 (4)	−0.0006 (4)
C13	0.0303 (6)	0.0296 (6)	0.0223 (6)	0.0006 (5)	0.0058 (5)	−0.0017 (5)
C14	0.0390 (7)	0.0282 (6)	0.0257 (6)	−0.0038 (5)	0.0128 (5)	−0.0048 (5)
C15	0.0496 (8)	0.0253 (6)	0.0321 (6)	0.0065 (6)	0.0189 (6)	0.0008 (5)
C16	0.0436 (7)	0.0323 (7)	0.0275 (6)	0.0111 (6)	0.0089 (5)	0.0055 (5)
C17	0.0414 (7)	0.0293 (6)	0.0218 (6)	0.0094 (5)	0.0077 (5)	−0.0015 (5)
C18	0.0691 (11)	0.0254 (7)	0.0611 (10)	0.0007 (7)	0.0090 (9)	−0.0098 (7)
C19	0.0657 (11)	0.0584 (10)	0.0316 (7)	0.0367 (9)	0.0033 (7)	−0.0055 (7)
C20	0.0749 (12)	0.0587 (11)	0.0495 (9)	0.0235 (9)	0.0391 (9)	0.0062 (8)
C21	0.0546 (9)	0.0442 (8)	0.0326 (7)	−0.0016 (7)	0.0122 (6)	−0.0157 (6)

### Geometric parameters (Å, º)

**Table:**

N1—C5	1.4680 (13)	C13—C14	1.3961 (18)
N1—C3^i^	1.4694 (17)	C13—H13	0.9500
N1—C1	1.4761 (17)	C14—C15	1.3874 (19)
N2—C1	1.4517 (17)	C14—C21	1.5159 (18)
N2—C2	1.456 (2)	C15—C16	1.3868 (19)
N2—C4	1.4615 (16)	C15—H15	0.9500
C1—H1A	0.9900	C16—H16	0.9500
C1—H1B	0.9900	C17—C20	1.533 (2)
C2—C3	1.510 (2)	C17—C19	1.5328 (19)
C2—H2A	0.9900	C17—C18	1.533 (2)
C2—H2B	0.9900	C18—H18A	0.9800
C3—H3A	0.9900	C18—H18B	0.9800
C3—H3B	0.9900	C18—H18C	0.9800
C4—H4A	0.9900	C19—H19A	0.9800
C4—H4B	0.9900	C19—H19B	0.9800
C5—H5A	0.9900	C19—H19C	0.9800
C5—H5B	0.9900	C20—H20A	0.9800
O1—C11	1.3760 (15)	C20—H20B	0.9800
O1—H1	0.88 (2)	C20—H20C	0.9800
C11—C16	1.3920 (18)	C21—H21A	0.9800
C11—C12	1.4086 (17)	C21—H21B	0.9800
C12—C13	1.4016 (16)	C21—H21C	0.9800
C12—C17	1.5353 (17)		
			
C5—N1—C3^i^	113.69 (9)	C14—C13—C12	123.91 (12)
C5—N1—C1	114.72 (9)	C14—C13—H13	118.0
C3^i^—N1—C1	114.05 (11)	C12—C13—H13	118.0
C1—N2—C2	114.42 (12)	C15—C14—C13	117.82 (11)
C1—N2—C4	115.31 (10)	C15—C14—C21	121.34 (12)
C2—N2—C4	114.44 (11)	C13—C14—C21	120.83 (12)
N2—C1—N1	119.16 (10)	C16—C15—C14	120.13 (12)
N2—C1—H1A	107.5	C16—C15—H15	119.9
N1—C1—H1A	107.5	C14—C15—H15	119.9
N2—C1—H1B	107.5	C15—C16—C11	121.33 (12)
N1—C1—H1B	107.5	C15—C16—H16	119.3
H1A—C1—H1B	107.0	C11—C16—H16	119.3
N2—C2—C3	117.06 (12)	C20—C17—C19	107.96 (14)
N2—C2—H2A	108.0	C20—C17—C18	110.29 (14)
C3—C2—H2A	108.0	C19—C17—C18	106.91 (14)
N2—C2—H2B	108.0	C20—C17—C12	109.72 (12)
C3—C2—H2B	108.0	C19—C17—C12	112.10 (10)
H2A—C2—H2B	107.3	C18—C17—C12	109.81 (12)
N1^i^—C3—C2	116.84 (11)	C17—C18—H18A	109.5
N1^i^—C3—H3A	108.1	C17—C18—H18B	109.5
C2—C3—H3A	108.1	H18A—C18—H18B	109.5
N1^i^—C3—H3B	108.1	C17—C18—H18C	109.5
C2—C3—H3B	108.1	H18A—C18—H18C	109.5
H3A—C3—H3B	107.3	H18B—C18—H18C	109.5
N2^i^—C4—N2	118.16 (16)	C17—C19—H19A	109.5
N2^i^—C4—H4A	107.8	C17—C19—H19B	109.5
N2—C4—H4A	107.8	H19A—C19—H19B	109.5
N2^i^—C4—H4B	107.8	C17—C19—H19C	109.5
N2—C4—H4B	107.8	H19A—C19—H19C	109.5
H4A—C4—H4B	107.1	H19B—C19—H19C	109.5
N1—C5—N1^i^	119.17 (14)	C17—C20—H20A	109.5
N1—C5—H5A	107.5	C17—C20—H20B	109.5
N1^i^—C5—H5A	107.5	H20A—C20—H20B	109.5
N1—C5—H5B	107.5	C17—C20—H20C	109.5
N1^i^—C5—H5B	107.5	H20A—C20—H20C	109.5
H5A—C5—H5B	107.0	H20B—C20—H20C	109.5
C11—O1—H1	110.4 (13)	C14—C21—H21A	109.5
O1—C11—C16	120.38 (11)	C14—C21—H21B	109.5
O1—C11—C12	119.23 (11)	H21A—C21—H21B	109.5
C16—C11—C12	120.39 (11)	C14—C21—H21C	109.5
C13—C12—C11	116.33 (11)	H21A—C21—H21C	109.5
C13—C12—C17	121.39 (11)	H21B—C21—H21C	109.5
C11—C12—C17	122.27 (10)		
			
C2—N2—C1—N1	81.95 (16)	C11—C12—C13—C14	0.39 (19)
C4—N2—C1—N1	−53.89 (17)	C17—C12—C13—C14	−179.70 (12)
C5—N1—C1—N2	−52.30 (16)	C12—C13—C14—C15	1.9 (2)
C3^i^—N1—C1—N2	81.31 (15)	C12—C13—C14—C21	−177.37 (13)
C1—N2—C2—C3	−67.63 (18)	C13—C14—C15—C16	−1.9 (2)
C4—N2—C2—C3	68.60 (19)	C21—C14—C15—C16	177.41 (14)
N2—C2—C3—N1^i^	−0.8 (2)	C14—C15—C16—C11	−0.4 (2)
C1—N2—C4—N2^i^	53.56 (9)	O1—C11—C16—C15	−178.23 (13)
C2—N2—C4—N2^i^	−82.27 (10)	C12—C11—C16—C15	2.9 (2)
C3^i^—N1—C5—N1^i^	−81.55 (10)	C13—C12—C17—C20	−115.74 (15)
C1—N1—C5—N1^i^	52.24 (8)	C11—C12—C17—C20	64.16 (17)
O1—C11—C12—C13	178.33 (11)	C13—C12—C17—C19	4.20 (19)
C16—C11—C12—C13	−2.75 (19)	C11—C12—C17—C19	−175.90 (14)
O1—C11—C12—C17	−1.58 (19)	C13—C12—C17—C18	122.88 (14)
C16—C11—C12—C17	177.35 (12)	C11—C12—C17—C18	−57.22 (17)

Symmetry code: (i) −*x*+1, *y*, −*z*+1/2.

### Hydrogen-bond geometry (Å, º)

*Cg*1 is the centroid of the C11–C16 ring.

**Table:**

*D*—H···*A*	*D*—H	H···*A*	*D*···*A*	*D*—H···*A*
O1—H1···N1	0.88 (2)	2.01 (2)	2.8534 (15)	161.6 (17)
C18—H18*B*···O1	0.98	2.30	2.966 (2)	124
C20—H20*A*···O1	0.98	2.41	3.058 (3)	124
C1—H1*A*···*Cg*1^ii^	0.98	2.90	3.851 (2)	163

Symmetry code: (ii) −*x*+1, −*y*+1, −*z*+1.
